# Animal models of critical care illnesses: current reality, future directions

**DOI:** 10.1186/s40635-025-00776-2

**Published:** 2025-06-25

**Authors:** John F. Fraser, Shigeaki Inoue, Yongming Yao, Marcin F. Osuchowski

**Affiliations:** 1 Critical Care Research Group, The Prince Charles Hospital, Brisbane, Australia; 2 Intensive Care Department, St Andrews War Memorial Hospital, Brisbane, Australia; 3 Department of Emergency and Critical Care Medicine, Wakayama Medical University, Wakayama, Japan; 4 Translational Medicine Research Center, Medical Innovation Research Division and Fourth Medical Center of the Chinese PLA General Hospital, Beijing, China; 5 Ludwig Boltzmann Institute for Traumatology, The Research Center in Cooperation With AUVA, Vienna, Austria

Critical care medicine addresses multiple life-threatening conditions including sepsis, polytrauma, shock and acute respiratory distress syndrome (ARDS) as well as recent global threats like COVID-19. Advancing clinical improvements for patients suffering from these illnesses is contingent upon understanding of their complex pathophysiology, developing precise/rapid diagnostic tools and effective therapies. Animal models have long served as a cornerstone of this endeavor offering invaluable discoveries including first test of closed-chest cardiac massage technique in 1959 (developed in dogs) and first use of anti-SARS-CoV-2 vaccine in 2020 (tested in rodents and non-human primates).

## The indispensable role of animal models

Animal models provide a controlled environment to dissect the intricate mechanisms underlying critical illnesses. Researchers can manipulate variables, observe disease progression, and test therapeutic interventions in ways impossible in human patients. For example, animal models of sepsis have been instrumental in elucidating the fluctuating dynamics of inflammatory mediators and the role of immunosuppression in sepsis pathophysiology [1]. Similarly, ARDS models have helped define the pathophysiology of lung injury and evaluate novel ventilation strategies [2]. In polytrauma research, animal models allow for controlled injury mechanisms and the study of systemic inflammatory responses [3]. During the COVID-19 pandemic, animal models rapidly provided essential data about viral pathogenesis, transmission, and potential therapeutic interventions [4].

## Translation gaps: a persistent challenge

Despite their acknowledged utility, many findings from animal studies fail to translate to human patients. Differences in physiology, genetics, and immune responses among species contribute to this translational gap [5]. The complexity of critical illnesses, often involving multiple organ systems and individual patient variability, further complicates the path to clinical applications. While some animal-based findings have led to clinical breakthroughs, their implementation typically suffers from long-term lags and many promising therapies tested in animals have failed in clinical trials. Furthermore, animal models rarely fully replicate the clinical syndrome being studied. Challenges such as reproducibility crisis and publication bias (towards positive results) further aggravate the problem.

## Public perception and ethical considerations

Animal-based experimentation faces increasing scrutiny from the lay public, driven by ethical concerns about animal welfare. Misinformation and emotional appeals often overshadow the scientific rationale and potential benefits of animal research [6]. Researchers need to be proactive in communicating transparently and to standardize the regulations that govern the care of animals. This will convincingly demonstrate that the animal research advancing critical care incorporates animal welfare as its central element—such initiatives have been already underway (e.g., www.eara.eu/patient-discovery) and should be reinforced.

## Charting the future: refining, replacing, and reimagining

While we (and many other scientists) believe that the need for animal models in critical care research is undeniable, it requires continuous, multi-directional adaptation and renovation. Key elements required for a superior translation of animal research to patients are listed in Table 1.

**Table 1:** Key subject areas in need of development to improve animal-to-human translation

• Advanced ICU-like features	• Expert inter-laboratory cooperation
• Large-species models	• Accessible animal model biobanks
• Population-like heterogeneity	• Tight integration with in vitro/silico/AI methods
• “Humanized” animal models	• Transparency & justification

As the critical care field has rapidly advanced over the last few decades, the role of an animal model as a sole, separate testing entity can be considered obsolete. In the modern version of critical care research, any animal model of disease should exist in an inter-connected, multimodal and mutually supportive research network. In such a network, standardization of models should be harmonized with data collection and biobanking. This, in long term, would enable more precise data comparison across many international centers increasing scientific reproducibility and robustness. In addition, artificial intelligence (AI) can enhance data analysis from complex animal models, identifying subtle patterns and predicting treatment responses with greater accuracy. Machine learning (ML) algorithms will optimize experimental designs, reducing the number of animals while maximizing the information gained.

Ultimately, a multi-faceted approach that combines refined animal models with advanced technologies and alternative methods [7] will better promote welfare concerns, reproducibility lapses and will be superior in advancing clinical improvements for patients. All those elements must be in turn supported by adequate, stable institutional funding and regulatory support.

## The objective of the special issue

This thematic collection will combine a palette of papers addressing the ongoing and expected contributions of animal models to critical care. Thanks to the guest editors from Australia, China and Japan, this special issue will also provide a unique perspective from the Asia–Pacific region. Irrespective of the geographical origins, however, we invite you to contribute your valuable experimental studies, especially those that combine translational–clinical insights. We also welcome your ideas (and works) on developing new critical care models and refining existing ones including AI/ML tools. Finally, we encourage the authors to prophesize future directions and challenges that the critical care field is going to face in the coming decade(s). This collection aims at deepening our existing knowledge and challenges us to cross new modeling frontiers. Its primary goal, however, is to promote progress in the care of patients in critical conditions.

## Data Availability

Not applicable.

## Abbreviations

ARDS: Acute respiratory distress syndrome
AI: Artificial intelligence
COVID-19: Coronavirus disease 2019
ML: Machine learning
